# Tracking Extracellular Matrix Remodeling in Lungs Induced by Breast Cancer Metastasis. Fourier Transform Infrared Spectroscopic Studies

**DOI:** 10.3390/molecules25010236

**Published:** 2020-01-06

**Authors:** Karolina Chrabaszcz, Katarzyna Kaminska, Karolina Augustyniak, Monika Kujdowicz, Marta Smeda, Agnieszka Jasztal, Marta Stojak, Katarzyna M. Marzec, Kamilla Malek

**Affiliations:** 1 Faculty of Chemistry, Jagiellonian University, 30-387 Krakow, Poland; karolina.chrabaszcz@doctoral.uj.edu.pl (K.C.); karolinakatarzyna.augustyniak@student.uj.edu.pl (K.A.); monika.kujdowicz@uj.edu.pl (M.K.); 2 Jagiellonian Centre for Experimental Therapeutics, Jagiellonian University, 30-384 Krakow, Poland; katarzyna1.kaminska@uj.edu.pl (K.K.); marta.wojewoda@jcet.eu (M.S.); agnieszka.jasztal@jcet.eu (A.J.); marta.stojak@jcet.eu (M.S.); katarzyna.marzec@jcet.eu (K.M.M.); 3 Department of Pathology, Medical Faculty, Jagiellonian University Medical College, 31-531 Krakow, Poland

**Keywords:** FTIR imaging, extracellular matrix remodeling, fibrous proteins, cancer metastases

## Abstract

This work focused on a detailed assessment of lung tissue affected by metastasis of breast cancer. We used large-area chemical scanning implemented in Fourier transform infrared (FTIR) spectroscopic imaging supported with classical histological and morphological characterization. For the first time, we differentiated and defined biochemical changes due to metastasis observed in the lung parenchyma, atelectasis, fibrous, and muscle cells, as well as bronchi ciliate cells, in a qualitative and semi-quantitative manner based on spectral features. The results suggested that systematic extracellular matrix remodeling with the progress of the metastasis process evoked a decrease in the fraction of the total protein in atelectasis, fibrous, and muscle cells, as well as an increase of fibrillar proteins in the parenchyma. We also detected alterations in the secondary conformations of proteins in parenchyma and atelectasis and changes in the level of hydroxyproline residues and carbohydrate moieties in the parenchyma. The results indicate the usability of FTIR spectroscopy as a tool for the detection of extracellular matrix remodeling, thereby enabling the prediction of pre-metastatic niche formation.

## 1. Introduction

Cancer malignant behavior can be terminal. Tumors can spread into organs and invade nearby and further tissues. The leading cause of death in advanced stages of cancer is metastasis formation and its consequences [1]. Several steps define metastasis. It starts from biochemical changes in the cancer cells and primary tumor niche, the production of chemokines, immune system reactions, and the destruction of healthy tissues by enzymes. Then the cancer process encompasses the whole body. The subsequent metastatic steps include intravasation into the blood and lymphatic vessels, transport, extravasation into distant sites, and colonization to form micrometastases and macrometastases [1,2]. Despite the rapid development of diagnostic techniques, particularly radiology (e.g., magnetic resonance imaging, positron emission tomography, X-ray), cytology, histopathology, and molecular methods, sensitivity in the detection of micrometastases and defining the pre-metastatic niche is not sufficient.

Recent studies have also introduced the concept of the pre-metastatic niche. This is the formation of a specialized microenvironment through extracellular matrix remodeling, immunosuppression, inflammation, and vascular hyperpermeability, created by the primary tumors in secondary organs and tissue sites for subsequent metastases [3,4]. Therefore, the characterization of the metastatic process before micrometastases formation is vital to supporting appropriate treatment and to decrease the cancer-related death rate.

Cancer-related processes transform several tissue structures [5,6]. Abnormal changes were found in the amount and composition of the extracellular matrix (ECM) in the primary tumor tissue and metastasis places, particularly in the lungs [6]. ECM remodeling is directly linked to tumor progression in the lungs [7]. ECM is a three-dimensional, non-cellular macromolecular network composed of three major components: proteins, glycosaminoglycans, and glycoconjugates. Changes in ECM architecture are always present during cancer development [7,8]. The remodeling process is associated with interactions between components of the cellular microenvironment such as hyaluronan, fibronectin [9], tenascin-C [10], laminin-322 [11], thrombospondin-1 [12], and collagens [9]. These proteins directly regulate the cellular functions, growth, differentiation, adhesion, migration, invasion, and colonization of cancer cells [9]. Furthermore, they can create a favorable environment for tumor survival that leads to disorders of tissue function.

Owing to the complicated mechanisms of ECM-cancer interactions, the remodeling process requires the application of sensitive and non-destructive methods. This is required to recognize not only its impact on the chemical composition of the entire tissue but also to define tissue sub-structures and their distribution within lung parenchyma. Fourier transform infrared (FTIR) spectroscopy is one of the techniques that allow the determination of a panel of biochemical changes occurring in tissue using a specific pattern of IR bands attributed to the most significant biological components such as proteins, lipids, carbohydrates, and nucleic acids. This method was successfully applied in the discrimination of healthy tissues from advanced cancer at various organs, collected as biopsies from clinical patients. The most prominent changes caused by cancer development in the breast [13] and esophagus [14] were an increase in lipids (2800–3000 cm^−1^) and DNA IR absorptions (1090–1080 cm^−1^) accompanied by an intensity decrease in the protein range (1800–1500 cm^−1^). In turn, studies of lung cancer via FTIR spectroscopy were conducted to establish the differences between cancerous and healthy lung tissue samples [15]; to investigate the spectral profile of malignant, adjacent to cancer, and healthy lung tissue [16]; to classify lung adenocarcinoma and its degree of malignancy [17]; to detect lung cancer from sputum samples [18].

To the best of our knowledge, no report in the literature investigates in detail the spectral and chemical transformations in the extracellular matrix due to cancer and metastasis progression using FTIR spectroscopy. Our previous studies, employing infrared spectroscopic imaging as the primary analytical tool, showed global chemical changes appearing during the neoplastic process in the lung due to metastasis from breast adenocarcinoma and identified small clusters of cancer cells settled in various lung environments [19]. Moreover, it was possible to identify the type of metastases, i.e., the attack of cancer cells through blood vessels or pleura, and to provide insight into their spectral heterogeneity [20]. We also provided FTIR-based discrimination of the early- and late-phases of cancer and metastasis development in blood plasma [21].

A few reports already showed the ability of FTIR spectroscopy to investigate protein structures (mainly collagen) and their changes in tissues induced by remodeling. For instance, a brain tumor can be discriminated from healthy tissue by the relative contribution of triple helices, β-sheets, and β-turns. The triple helix absorption at 1637 cm^−1^ was proposed as the indicator of collagen contents, but no intensity changes in this band were found in healthy, solid tumor, and diffuse tumor tissues [22,23]. Next, extracellular matrix remodeling was characterized spectroscopically by Bromberg and co-workers in the cardiomyopathic Syrian hamster heart [24]. The IR profile of healthy cardiac tissue differed from myocardial dysfunction in the collagen spectral region. In particular, decreased intensities of the amide III (1335–1201 cm^−1^), C-O carbohydrate residues bands (1031 and 1081 cm^−1^), as well as a lower ratio of the 1162 and 1172 cm^−1^ bands (C-OH stretches of hydroxyproline) were observed in the cardiomyopathic tissue. Another process, associated with extracellular component degradation, is fibrosis [25]. This process occurs as the final pathway of tissue damage in that mainly collagen I, III, and IV are degraded. Rapid quantum cascade laser IR (QCL-IR) imaging of renal transplant biopsies identified progressive fibrosis via determination of the intensity ratio of bands at 1035 and 1079 cm^−1^, assigned to collagen associated carbohydrate moieties (C–O and C–O–C stretches, respectively) [25].

The invasion of cancer cells in lung tissue and the formation of the pre-metastases niche is connected with changes in the structure and composition of fibrillar proteins, the main components of ECM [26,27]. Studies reporting investigations of this process indicated that there is a lack of tools to identify and predict the pre-metastatic niche formation, including ECM remodeling [28]. Therefore, to assess extracellular matrix remodeling caused by 4T1 breast cancer metastasis to the lung, we employed IR imaging, which permits the interrogation of tissue biochemistry with spatial information. The latter is an ex vivo orthotopic mouse model in which 4T1 breast cancer cells are injected into the mammary gland where the tumor grows and then metastases to the lungs within 2–3 weeks [29,30]. Our previous experiments showed how difficult it is to detect metastases in the very early phases of the disease based only on medical parameters such as lung weight, primary tumor volume, and the number of pulmonary metastases [29,30]. Here, we show the progression of chemical changes in lung regions not directly affected by the presence of secondary tumors within five weeks of the pulmonary breast cancer metastasis. We demonstrated, for the first time, utilization of FTIR spectroscopic imaging to study different fractions of the extracellular matrix to understand the process of the metastatic niche and provide the characteristics of remodeling in the lungs affected by metastasis.

## 2. Results and Discussion

### 2.1. Morphological Characterization of Lungs in Metastasis Development

The development of lung metastases was examined between the first and fifth weeks after cancer cell injection into the mammary gland, and it was compared to healthy control. A schematic presented in Figure S1 in Supplementary Materials reflects the main morphological and biochemical changes occurring in mice lungs during the pulmonary breast cancer progression in this specific animal cancer model [29,30,31,32,33].

**Figure 1:**
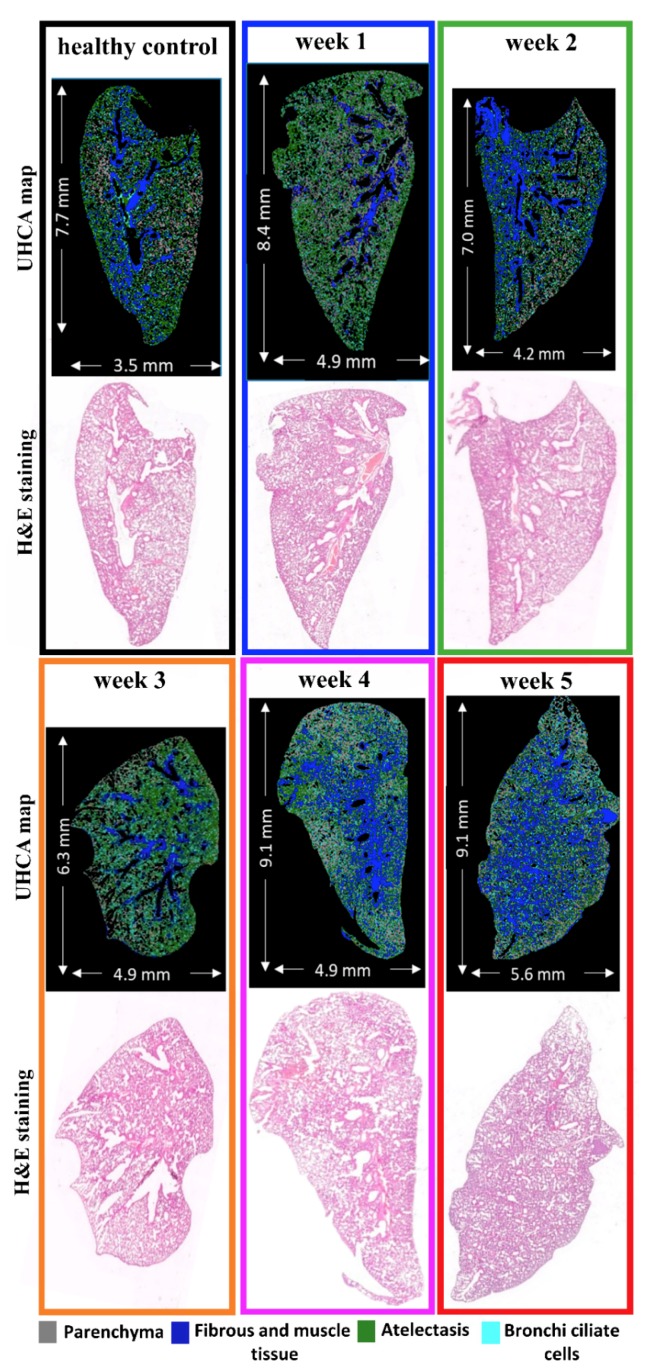
Comparison of false-color unsupervised hierarchical cluster analysis (UHCA) maps with hematoxylin and eosin (H&E) microphotographs collected from lung cross-sections. UHCA classification of the most abundant tissue types in lungs: parenchyma (grey), atelectasis (green), fibrous/muscular tissue (blue), and bronchi ciliated cells (aqua) corresponds to tissue structure observed in the H&E images. Magnified microphotographs after H&E staining with marked tissue types are shown in Figure S2 in Supplementary Materials.

According to hematoxylin and eosin (H&E) staining, the lungs on week 1 after 4T1 cells inoculation did not differ from the healthy control (Figure 1). In the early pre-metastatic phase (week 2), the lungs displayed histopathological signs of inflammation caused by granulocyte infiltration (Figure 1). In the third week, a single cancer cell or up to 10 cell clusters occurred in the lungs. No metastases or a maximum of two metastases per animal were observed under such conditions in the whole left lung (Table 1). In this phase, the weight of lungs slightly increased while a mass of the primary tumor in the breast increased tenfold (Table 1). Owing to further metastasis progression, we observed large, clear-cut metastatic foci in the lungs and visible scattered macrometastases. These occupied the lung parenchyma and were also found under the pleura (week 4, Table 1). In the last phase (week 5), the lungs were occupied by very advanced macrometastases of different sizes. Between 7 and 89 metastases were observed in the lungs of mice studied at this stage of the cancer progression (Table 1).

Lung lobes, in which no metastases were detected, were selected in this work to evaluate the remodeling of their morphology and biochemistry (Figure 1). We excluded tissue cross-sections with secondary tumors to avoid the effect of tumor metabolism on the surrounding lung cells. As shown in Figure 1, we assessed EMC in four types of tissue morphology typical for lungs, i.e., the main component of the lung, parenchyma. It is transformed into atelectasis in the late phase, as well as fibrous and muscle cells merged, and bronchi ciliated cells.

Normal lung parenchyma consists of airways and thin-walled alveoli (Figure 1 and Figure S2 in Supplementary Materials, gray class). The alveoli are composed of a single layer of squamous epithelium and are lined almost exclusively by type I and II pneumocytes. A thin layer of connective tissue and numerous capillaries can also appear between the alveoli structures. Pneumocytes type 2 produces surfactant which is stored in structures called lamellar bodies, and they release surfactant into the alveoli. Under normal conditions, a few alveolar macrophages trundle around the alveoli scavenging for debris that was not removed by the mucociliary apparatus.

Atelectasis of lung parenchyma results from the collapse of a part of the lung due to a decrease in the amount of air in the alveoli (Figure 1 and Figure S2 in Supplementary Materials, green class). The pathologist recognized the first signs of atelectasis in the lung parenchyma as already being in the control tissue. Atelectasis could occur in the control animals as a result of the collapse of the part of the lung due to inflammation caused by a factor different from cancer cells. In the second week, the atelectasis of lung parenchyma was more advanced. A visual inspection of the macro-metastatic phases (weeks 3 and 4) suggested that the atelectatic changes were similar. In the fifth week, the atelectasis occupied almost the entire cross-sections of the lung (Figure 1 and Figure S2 in Supplementary Materials). As expected, the increase of the atelectasis area was associated with an increase in lung weight (Table 1).

Airways are surrounded by connective tissue consisting of fibers, ground substance, and cells. This tissue contains bronchial arteries, venous trunks, lymphatics, and nerves. In the airways, smooth muscles are found in many structures. Thus, fibrous and muscular tissue mainly surround blood vessels and large bronchioles in the lung lobes (Figure 1 and Figure S2 in Supplementary Materials). The trachealis smooth muscle bridges the gap between the free ends of C-shaped cartilages at the posterior border of the trachea. Every single bronchiole has a complete fibromuscular wall with the muscle arranged in a spiral, while smaller bronchi possess only smooth muscle fibers. The tunica media of the pulmonary veins is constituted by cross-striated muscle tissue, which consists mostly of actin filaments in association with myosin [34]. Larger elastic pulmonary arteries are composed of multiple layers of the elastic lamina and smooth muscle cells. In our study, fibrous and muscle tissue was merged into one class (Figure 1, blue class) [5,35]. Damage of lung parenchyma caused by cancer progression may cause pulmonary fibrosis. This process is characterized by the accumulation of excess fibrous connective tissue which leads to changes in the normal architecture and function of the tissue. The increase in fibrous and muscle tissue in this study was observed in the fourth and fifth weeks after cancer cell inclusion.

The surface epithelium of bronchi and bronchioles contains two principal cell types: ciliated and secretory cells. Secretory cells based on their microscopic appearance can be divided into subtypes called Clara, goblet, and serous cells. In turn, the bronchi ciliated cells are formed by the congregation of microtubules associated with dynein arms to form axonemes of mobile cilia (Figure 1 and Figure S2 in Supplementary Materials) [36]. In our study, this class did not change with cancer progression.

### 2.2. FTIR-Based Discrimination of Chemical Changes in Lung Tissue Structures due to Metastasis

**Figure 2:**
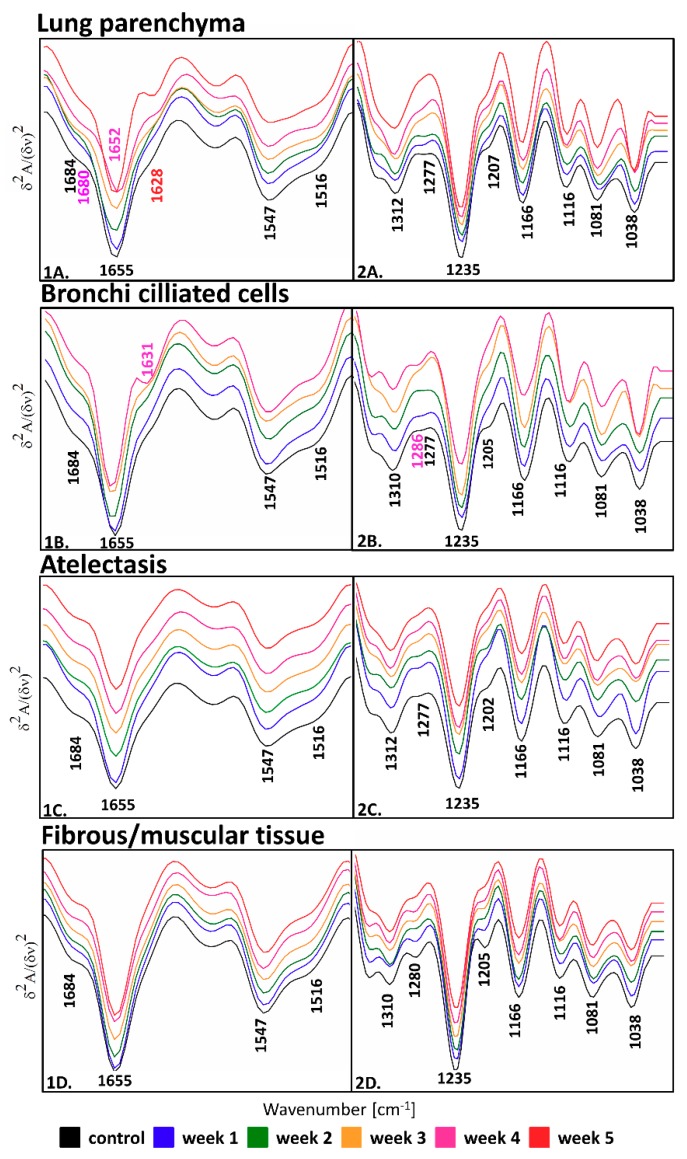
The comparison of second derivative IR spectra extracted from UHCA analysis for lung parenchyma (averaged from *n* = 37 spectra) (**A**), bronchi ciliated cells (averaged from *n* = 23 spectra) (**B**), atelectasis (averaged from *n* = 43 spectra), (**C**) and fibrous/muscular tissue (averaged from *n* = 44 spectra) (**D**). Spectra are shown in the regions of 1700–1480 (**1**) and 1340–1000 cm^−1^ (**2**). On week 5, bronchi ciliated cells were not found since atelectasis covers the entire area of the lungs.

With the use of FTIR spectroscopic imaging with a pixel size of 5.5 µm^2^, we collected chemical images of entire lung cross-sections. Since we collected a large spectral database (Table S1 in Supplementary Materials), we employed unsupervised hierarchical cluster analysis (UHCA) to differentiate tissue cross-sections into four assigned to the cell phenotypes described above (Figure 1 and Figure S3 in Supplementary Materials). Figure 2 shows the mean second derivative IR spectra extracted from the UHCA analysis from the corresponding classes of the cell phenotypes. The assignment of the UHCA classes to the cells was confirmed by the examination of lung morphology revealed by the H&E staining (Figure 1). Bands assignment observed in the fingerprint region of the IR spectrum is summarized in Table 2.

Alternation of intensity and shape of bands in the region of 1700–1485 cm^−1^, which were attributed to amide I and II vibrations of proteins, indicated changes in the secondary conformations of proteins. Apart from the 1655 cm^−1^ band assigned to α-helical secondary structures, the IR spectra of lung parenchyma and bronchi ciliated cells, which sweep small particles polluted bronchi, showed an increase in intensity at ca. 1630 cm^−1^ due to metastasis progression. This spectral feature was pronounced in the phase when the cancer cells clustered into large macrometastases, week 4 in Figure 2(1A,B). Macrometastases can obstruct bronchi (from tissue pressure or intrabronchial mass) leading to hindered airflow and damaged ciliated cells. In addition, the IR spectra of parenchyma exhibited variations in the position and shape of the spectral region below 1670 cm^−1^. The red-shift of the band at 1655 cm^−1^ by 3 cm^−1^ was observed bearing large metastatic nodules. This outcome suggests that proteins in the extracellular matrix of the lung parenchyma underwent proteolysis leading to the formation of β-sheet and β-turn conformations and the unwinding of collagen triple-helices [37]. In turn, atelectasis and fibrous/muscular structures did not show visible spectral changes in the amide I and II regions due to intravasation of the cancer cells to the lungs (Figure 2(1C,D)). In the case of fibrous/muscular tissue, the ca. 1630 cm^−1^ band was absent.

The most pronounced spectral changes associated with the ECM remodeling process were found in the region below 1400 cm^−1^ (Figure 2(2A–D)). Here, we observed bands attributed to the amide III mode of lung fibrillar proteins such as collagens [23], elastins [38], fibrillins, actins, and myosin (at ca. 1205, 1235, ca. 1280, ca. 1310, and 1318 cm^−1^) and glycoprotein- and collagen-associated carbohydrate moieties (at 1031 and 1081 cm^−1^), c.f. Table 2. Even though it is impossible to assign amide III bands to particular fibrillar proteins, the spectral changes in this region can indicate the remodeling of the extracellular matrix due to the expected degradation of elastin fibers and the action of metalloproteinases (MMP) degrading fibrillar proteins [30]. A visual examination of the IR spectra of the parenchyma and atelectasis classes showed the disappearance of the 1277 cm^−1^ band, contrary to the bronchi ciliated cells in that this band which was blue-shifted to 1286 cm^−1^ during the metastasis progression (Figure 2(2)). For atelectasis and fibrous tissue, this band remained unchanged in comparison to the control. The intensity of the bands at ca. 1205 cm^−1^ decreased in all investigated types of lung tissue due to the metastasis progression, except for the fibrous and muscular class (Figure 2(2)).

### 2.3. A Semi-Quantitative FTIR Approach in an Analysis of Proteins in the Lung Tissue Structures

**Figure 3:**
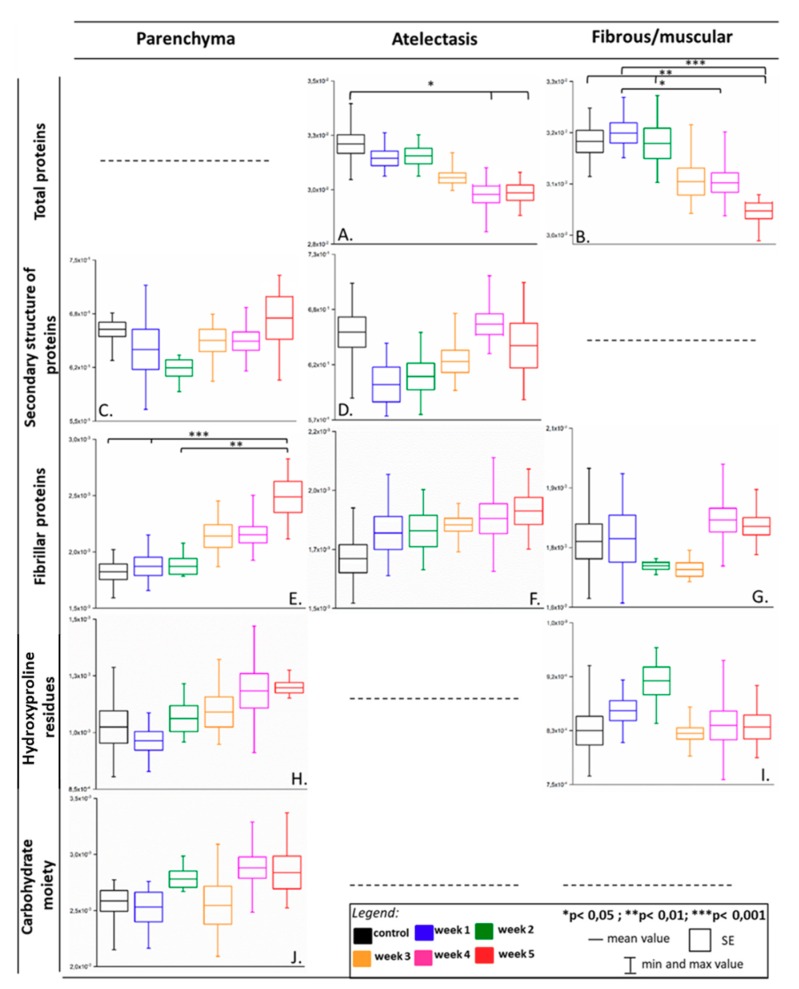
Box diagrams of integral intensities for the selected bands showing biochemical changes in parenchyma, atelectasis, and fibrous/muscular tissue in the metastasis progression in the lungs. Integration regions: total proteins [(1707–1608 cm^−1^) + (1589–1485 cm^−1^)], secondary structure of proteins [(1589–1485 cm^−1^)/(1707–1608 cm^−1^)], fibrillar proteins (1286–1193 cm^−1^), hydroxyproline residues (1187–1140 cm^−1^), carbohydrate moieties (1137–1015 cm^−1^). The bands’ assignment is given in Table 2.

In the semi-quantitative analysis, which gives a trend of molecular composition variation throughout metastasis development, we computed the integral intensities of the discussed-above bands for the classes of the lung parenchyma, atelectasis, fibrous, and muscular tissue since they represented the major components of the extracellular matrix (Figure 3).

The intensity sum of the amide I and II bands allowed the monitoring of changes in the total content of proteins (total proteins in Figure 3). Even though we observed changes in the secondary structures of proteins in the lung parenchyma, the content of proteins was constant for all phases in the disease progression (data not shown). Atelectasis, as well as fibrous/muscular tissue, significantly lost proteins in their chemical composition along with development of the first metastatic nodules in the lungs (week 3) and this process was continued till the macrometastatic phase (week 5) indicating that the proteolysis of proteins in the lung cells resulted from tumor metabolism (Figure 3A,B) [39]. An immunochemical analysis employed in a previous study on this cancer model assessed the expression of elastin and metalloproteinases MMP-2, MMP-4, and MMP-9 in the lung and it showed the degradation of elastin and increased expression of MMPs on weeks 3 and 4, respectively [30]. This result suggested that the intensities of amide I and II highlighted the degradation of proteins in the lungs, pointing out that this process was more advanced in dense structures of the tissue than in thin-walled alveoli.

To investigate changes in the secondary structures of proteins, a ratio of amide II to amide I was calculated (secondary structure of proteins in Figure 3). In this case, no changes were found in the class of fibrous and muscular tissue (data not shown) as expected from the visual inspection of its IR spectra. Generally, the trend of changes was similar for both the lung parenchyma and atelectasis (Figure 3C,D). A significant decrease of the amide II to amide I ratio was observed in the first week after 4T1 cell transplantation for the atelectasis tissue, even though H&E staining did not indicate any morphological differences between this time point (week 1) and the healthy control. Afterward, the process of protein remodeling was gradually enhanced until the macrometastatic phase, indicating the disturbance of tissue homeostasis. This process could be related to the formation of amyloid-like structures. This observation was congruent with the appearance of the shoulder at 1628 cm^−1^ in the secondary derivative IR spectra, as discussed above (Figure 2(1A,C)). Amyloid fibrils are formed from cross-β polymerization of β-pleated sheets, and it has been observed in various types of cancer [40]. Some reports discussing this issue showed that a large number of amyloid proteins were stored in dormant cancer cells and the amyloid-β precursor protein was engaged in cell adhesion molecules to bind with the ECM components [41]. Since the metastatic 4T1 cells used in this work also exhibited a high expression of the amyloid-β precursor protein, we hypothesized here that the observed changes in the secondary conformations of proteins could be associated with such transformation of these macromolecules [30,42]. Interestingly, we showed the elevated formation of β-sheets in the FTIR spectra of murine plasma derived from the same animal experiment [21]. Indeed, similar observations in the lung and plasma suggested that the IR spectra of blood plasma reflected processes of matrix remodeling in the metastatic lung.

The most prominent markers for evaluating extracellular remodeling are amide III bands assigned to fibrillar proteins (ca. 1281, 1235, and 1205 cm^−1^) [23]. Graphs in Figure 3E–G show various spectral responses in the content of these proteins in the selected lung morphological structures of 4T1 breast cancer-bearing mice. Local deposition of fibrillar proteins in the lung parenchyma began at the micrometastasis stage (week 3) and exhibited the highest level for lungs with advanced metastases (week 5), see Figure 3E. According to Bromberg and co-workers [24], we explicitly correlated this funding to collagen remodeling in the extracellular matrix. This was because other spectral markers of collagens, i.e., at 1166 cm^−1^ (hydroxyproline residues) and in the region of 1137–1015 cm^−1^ (carbohydrate moieties), showed trends of changes similar to the region of the amide III bands (Figure 3H,J). The class of atelectasis with features of fibrotic structure exhibited variations in the intensity of the amide III bands (Figure 3F). In the early stage, in the first week of cancer cell transplantation, there was a mild deposition of fibrillar proteins that was maintained until the terminal phase of cancer progression. Since no intensity changes were found for the two collagen bands below 1200 cm^−1^, the observed immunochemical degradation of elastin and the activity of the MMPs could be reflected by the spectral variation in the amide I, II, and III regions of the lung atelectasis [30]. Fibrous and muscular tissue surrounds blood vessels and large bronchioles and it is composed of collagenous fibers and actin filaments, where it showed a decrease of fibrillar proteins in the pre- and early-metastasis stage (week 2 and week 3), contrary to the remaining cancerous phases (Figure 3G). This result could suggest that the IR spectra of this lung structure indicated remodeling of the vascular walls due to their enhanced permeability for cancer cells circulating in the blood system at the early phase of cancer metastasis [43]. This process must be accompanied by the structural deformation of proteins around the hydroxyproline residues, which gradually occurs in the pre-metastatic phase, see graph in Figure 3I.

Finally, we correlated the spectral parameters extracted from each cross-section to a mass of the lungs in the investigated animals using the Spearman correlation test (Table 3). The Spearman’s rank correlation coefficients (R), as well as values of probability (P), suggested that the observed molecular changes in structure and composition of the lung proteins may correlate with physical changes of the metastatic organ. Further investigations on a more extensive set of samples must be performed.

## 3. Materials and Methods

### 3.1. Sample Preparation and Histological Analysis

Lung tissue samples were taken from inbred mouse strains BALB/cAnNCrl (control) and BALB/cAnNCrl within five weeks (weeks 1, 2, 3, 4, and 5) after orthotopic inoculation with viable 4T1 tumor cells of metastatic breast cancer. In total, six experimental groups (*N* = 18 animals in total, *N* = 3 per each group). Isolated lungs were washed in saline and fixed with 4% formalin buffered solution for 48 h. Paraffin-embedded cross-sections of tissues were cut from the middle of the isolated left lung (Table S1 in ESI). Then, 7 μm-thick cross-sections were prepared using a paraffin method on an Accu-Cut^®^ SRM™ 200 Rotary microtome mounted on CaF_2_ windows and dewaxed before FTIR imaging. This fixation method optimally preserves the structure of the tissue required for histological analysis by blocking autolysis and by crosslinking the amine groups of proteins. Despite the impact of the fixative agent on the tissue component, these alternations were smaller than the molecular changes associated with disease and did not affect spectral differentiation [44,45]. After the collection of the FTIR images, the same sections were stained with hematoxylin and eosin (H&E) for gold-standard histopathological examination. Digitized photographs were taken from the H&E slides. The examination and photographic documentation were performed using an Olympus BX53F white-light microscopic equipped with a DP74 digital camera. All investigations presented in this work conformed to the Guide for Care and Use of Laboratory Animals published by the US National Institutes of Health. A local animal research committee approved the experimental procedures used in the present study (permit no. LKE140/2013).

### 3.2. An Assessment of the Primary Tumor, Number, and Type of Metastases in the Lungs

Primary tumors were carefully dissected from the surrounding tissue and weighed. Isolated lungs were washed in saline, weighed, and fixed with 4% formalin buffered solution. To count the number of metastatic sites, we cut the lungs into lobes and the pulmonary metastases were counted on the surface of the lung lobes under a magnifying glass.

### 3.3. FTIR Spectroscopic Imaging and Data Analysis

We used a combination of an Agilent 670-IR FTIR spectrometer and a 620-IR microscope working in rapid mode, which allows for the collection of 16,384 spectra from an area of ca. 495,616 μm^2^ within 90 s (Santa Clara, CA, USA). A focal plane array (FPA) detector cooled with liquid nitrogen was coupled with this equipment. The detector consisted of a matrix of 16,384 pixels, arranged in a 128 × 128 grid format. The collection of IR images was performed in transmission mode. FTIR spectroscopic imaging used a 15× Cassegrain objective and condenser optics with NA of 0.62 and a projected FPA pixel size of 5.5 μm × 5.5 μm, giving a measured area of ca. 704 μm × 704 μm. All FTIR spectra were recorded by co-adding of 64 scans and in the range of 3800 to 900 cm^−1^ with a spectral resolution of 8 cm^−1^.

Pre-processing of the FTIR spectra and chemometric analysis were performed using CytoSpec (ver. 2.00.01) [31], MatLab (R2015a, Natick, MA, USA), and Origin 9.1 (ver. 2018b, OriginLab, Northampton, MA, USA) software. Firstly, a quality test was employed to introduce a threshold level and to eliminate signals with an absorbance lower than 0.2 and greater than 1.2. This operation was performed in the region between 1620 and 1680 cm^−1^. To remove spectral noise, we executed PCA-based noise reduction with 15 PCs. Resonant Mie scattering EMSC correction using seven principal components was performed on all spectra [32]. Next, second derivative IR spectra were calculated with 9 smoothing points according to the Savitzky–Golay protocol. All spectra were then vector normalized in the 914 to 1770 cm^−1^ region to avoid differentiation to the sample thickness. Unsupervised hierarchical cluster analysis (UHCA) was executed in the spectral region of 970 to 1770 cm^−1^ using the second derivative FTIR spectra. Spectral distances were computed as D-values and the individual clusters were extracted according to Ward’s algorithm. Four classes were selected to differentiate the tissue types of interest.

Mean second derivative spectra were used for the calculation of the integral intensity of various bands using the OPUS 7.0 program (Bruker Optics, Bullerica, MA, USA, Version 7.2.139.1294). For this purpose, a linear baseline was drawn through the peak edges, and the spectrum below this line was integrated over the wavenumber range of the band. Box plots were constructed for an analysis of variance performed using the statistical model (ANOVA) in the OriginPro 9.1 software. Tukey’s test was employed to compute significance values *p*. The correlation between the lung-to-body weight ratio [%] and selected FTIR biomarkers was performed via the Spearman’s rank correlation coefficient (R) and probability (P) in SPSS Statistics 24 (IBM Corporation, New York, NY, USA) software.

## 4. Conclusions

This study comprehensively described changes in proteins of the extracellular matrix in the lungs as being the primary site of metastasis in the mice model of breast cancer. Using large-area scanning implemented in FTIR spectroscopic imaging indicated molecular changes across whole cross-sections of the lungs giving insight into the separated tissue structures. We found that using FTIR spectroscopy; we obtained the biochemical information that allowed us to differentiate not only the type but also the degree of protein degradation. The spectral changes that appeared in lung parenchyma and atelectasis indicated a significant variation in secondary conformations of proteins and their composition. The former appeared similarly in both main constituents of the lung but the deposition of fibrillar proteins was specific for each of them. These alternations occurred before the invasion of breast cancer cells to the lungs suggesting that the amide I–III regions in the IR spectrum were sensitive to the signaling of the primary tumor. Consequently, this highlighted inflammation of the lung due to the transplantation of breast cancer cells recognized by H&E staining in the early phase and/or the formation of the ECM environment suitable for settling metastatic cancer cells.

## Figures and Tables

**Table 1:** The number of metastases, lung weight expressed as a percentage of body weight, and primary tumor mass in the 4T1 metastatic breast cancer model (*N* = 3 animals per experimental group).

Group	Number of Metastasis	Lungs Mass [% of Body Weight]	Primary Tumor Mass [g]
control	0	0.795–0.894	0
week 1	0	0.785–0.903	0
week 2	0	0.731–0.8	0–0.024
week 3	0–2	0.82–0.84	0.203–0.257
week 4	2–5	0.75–0.895	0.493–0.700
week 5	7–89	0.827–0.986	0.927–2.562

**Table 2:** An assignment of the major bands of Fourier transform infrared (FTIR) spectra depicted in Figure 2 [14,22,23,24,25].

Vibration Modes	Position [cm^−1^]	Assignment
ν(C=O), amide I: β-turns, β-sheets	1684, 1680	Proteins
ν(C=O), amide I: α-helices	1655, 1652	Proteins
ν(C=O); amide I, parallel β-sheets	1631, 1628	Proteins
δ(NH); amide II of proteins	1547	Proteins
in-plane δ(CH) of phenyl ring	1516	Tyrosine residues
ν(C-N)/δ(N-H)/ν(CH_3_-C): amide III	1310, 1312	Proteins
amide III	1286–1202	Fibrous proteins
ν(C-OH)	1166	Hydroxyproline residues
ν(C-O), δ(C-O-H), δ(C-O-C)	1116, 1081, 1038	Collagen-associated carbohydrate moieties

**Table 3:** The correlation between the lung-to-body weight ratio [%] and selected FTIR biomarkers associated using the Spearman’s rank correlation coefficient (R) and probability (P).

Spectral Parameter	Lungs Mass [% of Body Weight]	
	Spearman R	Spearman P
PARENCHYMA		
Secondary structure of proteins	0.82	0.001
Fibrillar proteins	0.81	0.001
Carbohydrates moiety	0.81	0.001
Hydroxyproline residues	0.84	0.0006
ATELECTASIS		
Total proteins	0.84	0.0006
Secondary structure of proteins	0.88	0.0002
FIBROUS/MUSCULAR		
Total proteins	0.87	0.0002
Fibrillar proteins	0.82	0.001
Hydroxyproline residues	0.82	0.0009

## Supplementary Materials

The following are available online, Table S1. Summary of collected data from *N* = 3 mice/group. Figure S1. A schematic of the investigated phases of pulmonary breast cancer metastasis. Development of lung inflammation and metastases were examined from week 1 to week 5 following 4T1 cancer cells transplantation. Figure S2. H&E microphotographs (20x) showing cellular phenotypes of lung parenchyma observed in healthy control (A) and advanced macrometastasis (B). Figure S3. UHCA clustering of the measured IR images of the lung cross-sections into parenchyma (grey), atelectasis (green), fibrous/muscular (blue), and bronchi ciliated cells (aqua) for pulmonary metastasis of breast cancer compared to the healthy control; weeks 1 and 2–the pre-metastatic phase, week 3–the early micrometastatic stage, week 4–the advanced micrometastatic phase, and week 5–the advanced macrometastatic stage. For more information about the FTIR imaged area of tissues and collected spectra, see Table 1.
