# Supplementary material for: Tracking Extracellular Matrix Remodeling in Lungs Induced by Breast Cancer Metastasis. Fourier Transform Infrared Spectroscopic Studies
Source: Molecules. 2020 Jan 6;25(1):236. doi: 10.3390/molecules25010236 (PMC6982691; doi:10.3390/molecules25010236)
Supplement: Supplementary file 1 [file molecules-25-00236-s001.pdf]

molecules

Supplementary Materials

Research Article

Tracking extracellular matrix remodeling in lungs induced by breast cancer metastasis. Fourier Transform Infrared spectroscopic studies

Karolina Chrabaszcz[1§], Katarzyna Kaminska[2§], Karolina Augustyniak[1], Monika Kujdowicz[1,3], Marta Smeda[2], Agnieszka Jasztal[2], Marta Stojak[2], Katarzyna M. Marzec[2], Kamilla Malek[1*]

[1] Faculty of Chemistry, Jagiellonian University, Gronostajowa 2, 30-387 Krakow, Poland
[2] Jagiellonian Centre for Experimental Therapeutics, Jagiellonian University, Bobrzynskiego 14, 30-384 Krakow, Poland
[3] Department of Pathology, Medical Faculty, Jagiellonian University Medical College, Grzegorzecka 16, 31-531, Krakow, Poland

molecules

Table S1. Summary of collected data from N=3 mice/group.

Group	No. of lung cross-sections	FTIR-imaged area [mm^2]	No. of recorded FTIR spectra
control	14	315.6	10 846 377
week 1	14	338.3	11 118 139
week 2	14	311.1	10 248 459
week 3	14	329.0	11 002 633
week 4	13	355.7	11 784 069
week 5	9	298.9	10 158 080

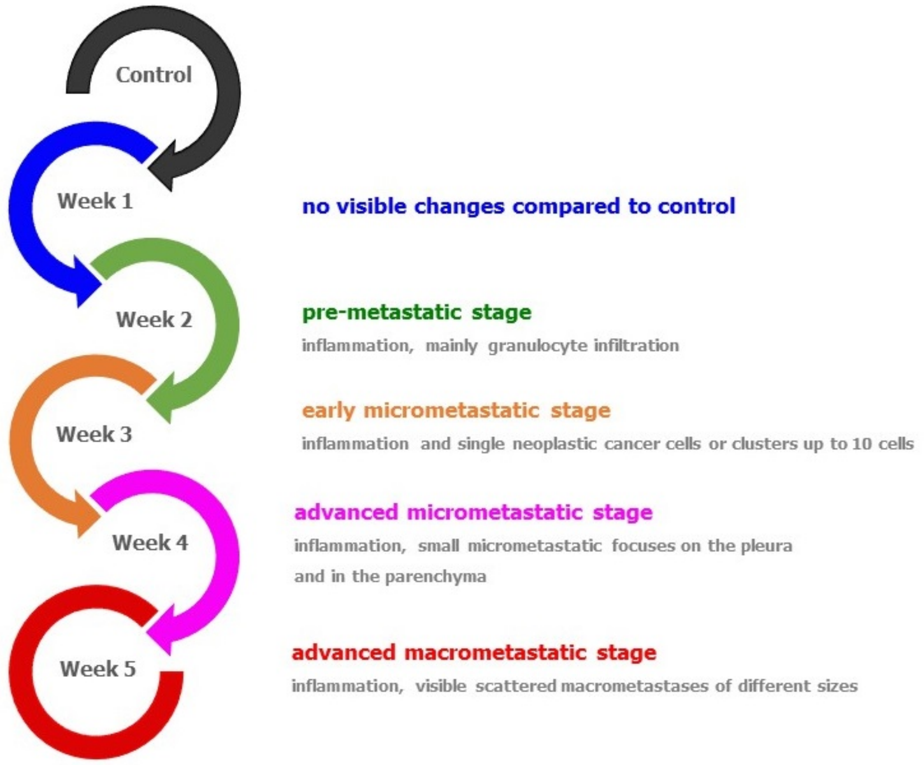

Figure S1. A schematic of the investigated phases of pulmonary breast cancer metastasis. Development of lung inflammation and metastases were examined from week 1 to week 5 following 4T1 cancer cells transplantation

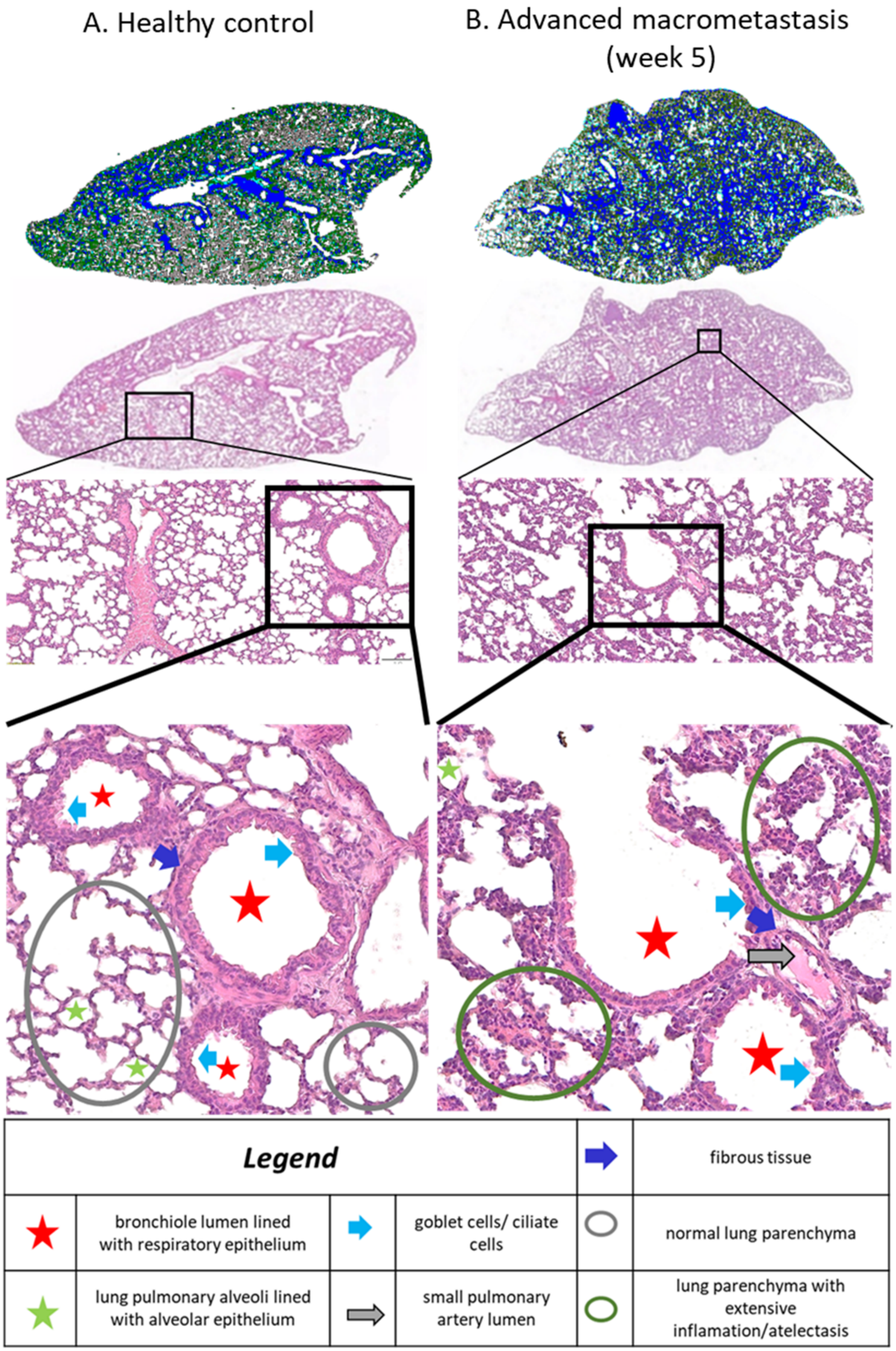

Figure S2. H&E microphotographs (20x) showing cellular phenotypes of lung parenchyma observed in healthy control (A) and advanced macrometastasis (B).

molecules
MDPI

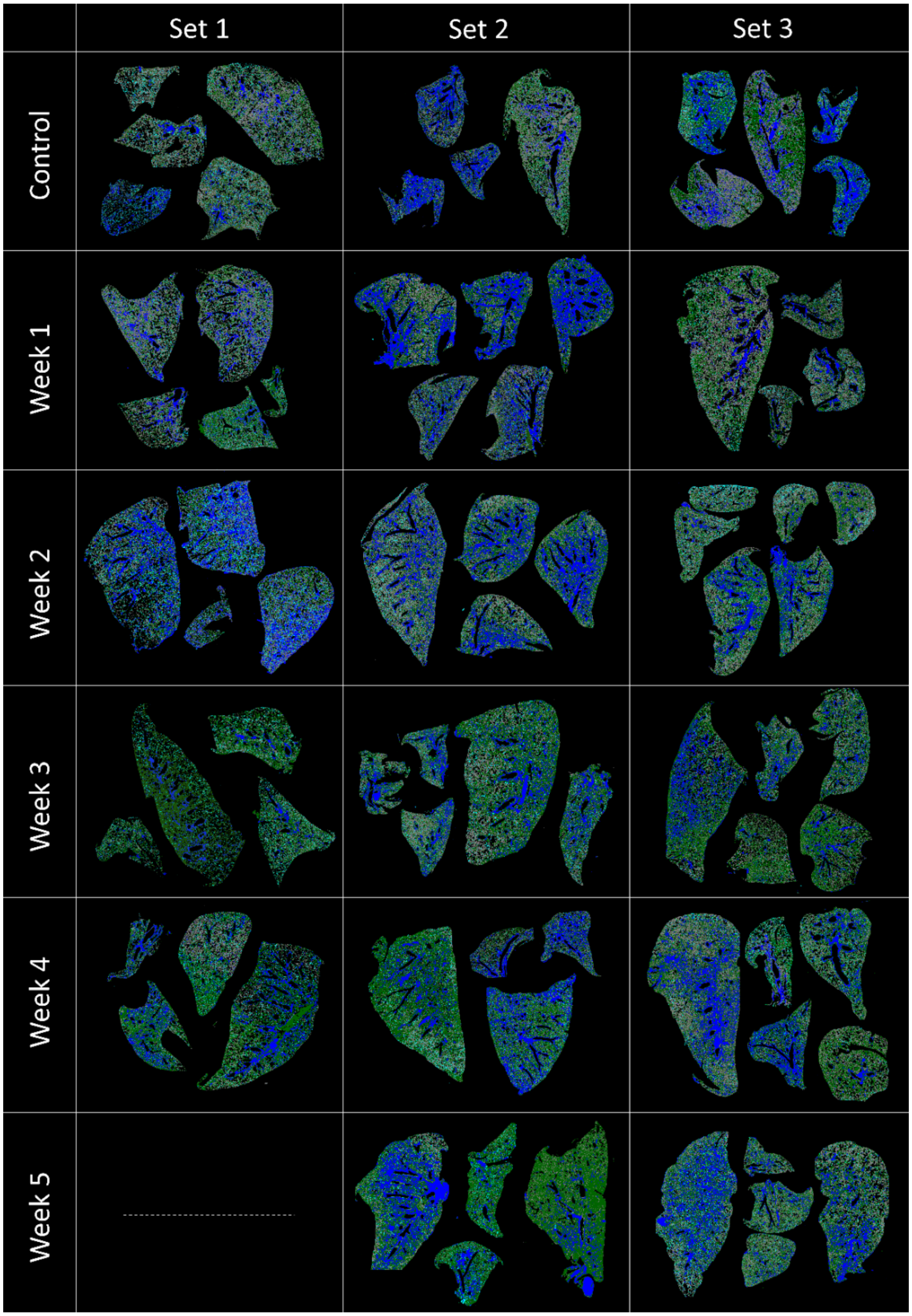

Figure S3. UHCA clustering of the measured IR images of the lung cross-sections into parenchyma (*grey*), atelectasis (*green*), fibrous/muscular (*blue*), and bronchi ciliate cells (*aqua*) for pulmonary metastasis of breast cancer compared to healthy control; week 1 and 2 – the pre-metastatic phase, week 3 – the early micrometastatic stage, week 4 – the advanced micrometastatic phase, and week 5 – the advanced macrometastatic stage. For more information about FTIR imaged area of tissues and collected spectra see Table 1.
